# Novel method for predicting nonvisible symptoms using machine learning in cancer palliative care

**DOI:** 10.1038/s41598-023-39119-0

**Published:** 2023-07-26

**Authors:** Kazuki Shimada, Satoru Tsuneto

**Affiliations:** 1 Department of Palliative Medicine, Kyoto University Hospital, 54 Kawaharacho, Shogoin, Sakyo-ku, Kyoto, 606-8507 Japan; 2 Department of Palliative Medicine, Graduate School of Medicine, Kyoto University, Kyoto University Hospital, 53 Kawaharacho, Shogoin, Sakyo-ku, Kyoto, 606-8507 Japan

**Keywords:** Cancer, Signs and symptoms, Machine learning

## Abstract

End-of-life patients with cancer may find expressing their symptoms difficult if they can no longer communicate verbally because of deteriorating health. In this study, we assessed these symptoms using machine learning, which has excellent predictive capabilities and has recently been applied in healthcare. We performed a retrospective clinical survey involving 213 patients with cancer from August 2015 to August 2016. We divided the reported symptoms into two groups—visible and nonvisible symptoms. We used decision tree analysis, an analytical machine learning method that organizes and analyzes information in the form of a tree diagram to visually represent the information structure. Our machine learning model used patient background data and visible symptoms to predict nonvisible symptoms: pain, dyspnea, fatigue, drowsiness, anxiety, delirium, inadequate informed consent, and spiritual issues. The highest and/or lowest values for prediction accuracy, sensitivity, and specificity were 88.0%/55.5%, 84.9%/3.3%, and 96.7%/24.1%, respectively. This work will facilitate better assessment and management of symptoms in patients with cancer. This study was the first to predict nonvisible symptoms using decision tree analyses for patients with cancer receiving palliative care. Notably, applications based on our results may assess symptoms to the same extent as healthcare professionals.

## Introduction

Palliative care has been developed in Japan primarily because of a policy focusing on cancer care^1^. Because of the increased life expectancy of patients with cancer, these individuals are no longer confined to inpatient settings during their illness^2^. The focus of palliative care for patients with cancer is shifting to general practice by healthcare professionals who do not specialize in this type of care. Although palliative care training is offered to healthcare professionals across Japan^3^, short-term training is insufficient^4^. Studies have reported that the availability of palliative care services is associated with general practitioner confidence and improved patient quality of life^5^.

Palliative care often begins with an assessment of symptoms that are known only to the patient and require a certain amount of time to evaluate. However, in general practice, the hectic and broad nature of care implies that the time to perform these detailed evaluations may be insufficient. Additionally, the hectic nature of general practice may result in a lower quality of clinical care; therefore, recent studies have aimed to support medical care by investigating clinical data using machine learning^6^. Recently, machine learning has been used to increase the diagnostic quality of imaging information, such as radiological images. The application of machine learning focuses on avoiding diagnostic errors in imaging and improving the diagnostic efficiency^7,8^. Therefore, machine learning could provide improved methods for assessing nonvisible symptoms in patients with cancer, improving the overall quality of healthcare, including palliative care, and better prognoses for such patients^9^.

End-of-life patients with cancer may have difficulty expressing their symptoms if they can no longer communicate verbally owing to the deterioration of their general condition^10^. When verbal communication with the patient is difficult, experience is required for assessing subjective symptoms known only to the patient^5^. Additionally, the number of palliative care specialists in rural areas is often limited^11^. Therefore, a supportive tool that can aid symptom assessment and management in cancer palliative care is greatly needed. This study aimed to create a model to predict nonvisible symptoms from visible symptoms and basic patient characteristics using machine learning.

## Materials and methods

### Data collection

We retrospectively collected patient data from three institutions located in Fukui Prefecture, Japan: University of Fukui Hospital, Fukui Prefectural Hospital, and Sugita Genpaku Memorial Obama Municipal Hospital (Supplementary Data 1). Although missing values must be addressed to create accurate machine learning models, we considered that deletion or completion of missing values may degrade the quality of training data when the number of data is small. We also considered that collecting good-quality data on large cases in the area of palliative care would be difficult, which is a common target for machine learning. Therefore, we retrospectively collected clinical data without missing values. In this study, 213 patients with cancer were included. Among the patients treated by the palliative care teams of the three aforementioned institutions, only those in which the first author was also involved were included in the analysis. The first author avoided personal bias in symptom assessment and other supportive activities by working collaboratively with the palliative care team of each facility (Supplementary Data 1). Both outpatients and inpatients were included in the study. No exclusion criteria based on patient background or disease was used. We collected patient characteristics via an initial assessment during palliative care team intervention, including symptoms and details of the palliative care team’s intervention. Patients were recruited for 1 year, and those who were included at the end of the recruitment period were observed for 28 days as follows.

To assess palliative team activities consistently across multiple institutions, appropriate formatting of palliative team activity records is necessary. Therefore, we used the previously published Standard Format for Palliative Care Team Activities 1.0 (SF-PCTA1.0) to collect and standardize activity records. The contents of SF-PCTA1.0 were divided into sections, as follows: Section I. Cover sheet, Section II. Reasons for referral and initial assessment, and Section III. Activities. The actual form and method for completing each item of the SF-PCTA1.0 are presented in Supplementary Data 2. The first author recorded the cover sheet, reasons for referral, and activities, referring to the original publication^12^. Two differences in the use of the SF-PCTA1.0 in this study compared with the original work were (i) that it was used in supporting activities at multiple sites and (ii) that the observation period and site name were added to the cover sheet.

Patients were enrolled over a 12-month period, and data were collected over a 13-month period beginning in August 2015, including the observation period of the last patient enrolled. In the study by Sasahara et al., to create the SF-PTCA1.0, the interventions of the palliative care team were described daily; however, the observation period was over a monthly basis. In this study, the observation period was also on a daily basis. Consistent with the original publication on the SF-PCTA1.0, the maximum observation period for a patient was 28 days^12^. Additionally, according to the original paper, the participants were patients who had been referred to the palliative care team for treatment, and the data were a simple aggregation of routine medical care^12^. As the first author is a consultant on palliative care in workplaces, he reviewed the records of all items of the SF-PCTA1.0 to avoid duplicate recommendations and implementations for the same item. To avoid bias because of the first author’s subjectivity in the SF-PTCA1.0 entry process, the activities conducted jointly by the first author and palliative care team at each facility were recorded directly in the SF-PTCA1.0. If any difficulty in determining the input was experienced, the appropriateness of the input was discussed with the palliative care team at each facility.

**Figure 1:**
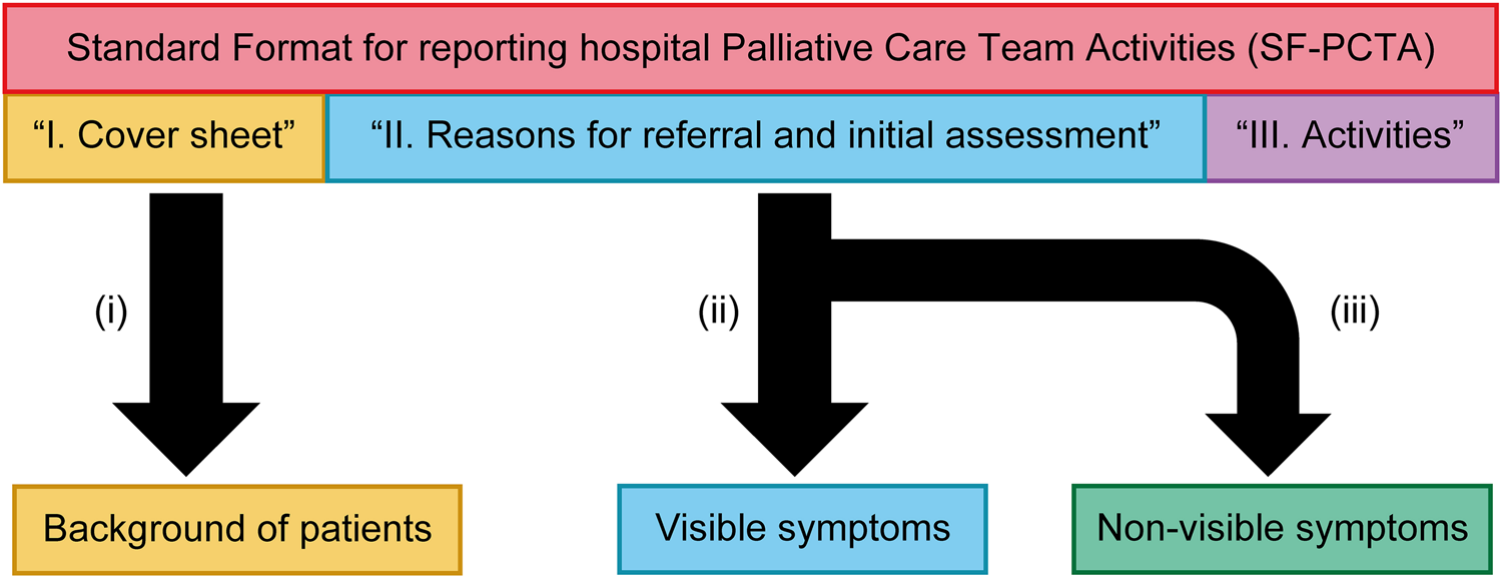
Data preprocessing. (i) In the process of changing from “I. Cover sheet” in the SF-PCTA1.0 to “Background of cancer patients,” the following processes were followed. Input variables were assigned for the categories under the place of medical treatment, age, sex, cancer site, status of anticancer treatment, Eastern Cooperative Oncology Group Performance Status (ECOG-PS), and referring a person. Cancer sites included in “other” were excluded. Real numbers were used for the variable representing age. Other variables were categorized as 1 for presence and 0 for absence. Patient outcomes, observation periods, and types of hospitals were excluded from the input variables for this study. (ii) Among the items included in “II. Reason for referral and initial assessment” in the SF-PCTA1.0, “visible symptoms” were defined as symptoms that could be easily assessed in our observation. Categorization was performed and rated 1 if the patient was presumed to have symptoms and 0 if the patient was presumed not to have symptoms. (iii) However, symptoms difficult to evaluate using general observation were designated as nonvisible symptoms. Categorization was performed and rated 1 if the patient was presumed to have symptoms and 0 if the patient was presumed not to have symptoms.

Our goal was to create a machine learning-based model to predict symptoms difficult to assess by general observation from patient characteristics and symptoms easy to assess. General observations were based on visual information, such as quantity and degree. For example, a small amount of bowel movements could be evaluated as constipation, and the frequent use of the toilet during the night, when the patient should be sleeping, could be evaluated as sleep disturbance. The behavior of receiving vomit in a cup while regurgitating saliva from the mouth could be rated as nausea. Abdominal distension can be assessed by visual examination with others. Even though general observations could not assess the intensity of the patient’s symptoms, they provided clues for others to assess the presence or absence of symptoms empirically via characteristic visual information. It is “difficult to evaluate by general observation” when information obtained through verbal communication, such as a medical interview, rather than information obtained through physical examination, such as visual examination, is the main cue for evaluation regarding the presence or absence of symptoms. Section I of the SF-PTCA1.0 was used as the source of patient characteristic data and section II was used as the source of symptom data (Fig. 1). We assigned patient characteristics as input variables, including the place of medical treatment, age, sex, cancer site within the body, status of anticancer treatment, Eastern Cooperative Oncology Group (ECOG) performance status, and referring person (Table 1). We also assigned visible symptoms as input variables, including a decrease in food intake, nausea, abdominal distension, constipation, edema, and sleep disturbances. We assigned the nonvisible symptoms as output variables, including pain, dyspnea, fatigue, drowsiness, anxiety, delirium, inadequate informed consent, and spiritual issues. The distinction between visible and nonvisible symptoms did not correspond to a medical definition, such as their subjective or objective symptoms, but was based on a simple assessment from a clinical perspective by healthcare professionals, patients, and their families.

**Table 1:** Contents of the variables.

Background of patients		Variables
Place of medical treatment	Hospitalization	Input
	Outpatient	
Age		
Sex	Male	
	Female	
Cancer site	Pancreas	
	Unknown	
	Lung	
	Breast	
	Head and neck	
	Biliary tract	
	Colon/rectum	
	Prostate	
	Under investigation	
	Kidney/bladder	
	Esophagus	
	Uterus/ovary	
	Liver	
	Stomach	
	Lymph node/hematology	
Status of anticancer treatment	No further anticancer treatment	
	Before anticancer treatment	
	Under anticancer treatment	
ECOG performance status		
Referring person	Doctor	
	Nurse	
Visible symptoms	Decrease in food intake	
	Nausea	
	Abdominal distension	
	Constipation	
	Edema	
	Sleep disturbance	
Nonvisible symptoms	Pain	Output
	Dyspnea	
	Fatigue	
	Drowsiness	
	Anxiety	
	Delirium	
	Spiritual issues	
	Inadequate informed consent	

### Study approval

This is a retrospective study and was conducted according to the Ethical Guidelines for Medical Research Involving Human Subjects issued by the Ministry of Education, Culture, Sports, Science, and Technology and the Ministry of Health, Labor, and Welfare (issued February 9, 2015 and revised March 31, 2015). This was approved by the institutional review board of The University of Fukui (Approval number: 20160011), Fukui Prefectural Hospital (Approval number: 16-10) and Sugita Genpaku Memorial Obama Municipal Hospital (Unnumbered). We did not obtain informed consent for data collection because the data were anonymized and we used existing materials and information. The above mentioned ethics committees’ waived the need of obtaining informed consent since the study was a simple accumulation of routine medical care. We followed the Declaration of Helsinki in collecting patient data, and the study was ethically reviewed and accepted by the three participating institutions.

### Data preprocessing

The overview of data preprocessing is shown in Fig. 1. We obtained patient characteristics from Section I of the SF-PCTA1.0. We excluded data from the cover sheet as input data because the information would be used for a timeframe after the time of symptom assessment. The University of Fukui Hospital and Fukui Prefectural Hospital specialize in cancer care. Sugita Genpaku Memorial Obama Municipal Hospital is engaged not only in cancer care but also in various other medical services as a central public hospital in the region. Institutional information about the presence or absence of specialized cancer care was not associated with the frequency of “reason for referral” and “problem identified by the first author” in symptom assessment (Supplementary Data 3). Therefore, we excluded institutional information from the input data. For patients in whom the cancer site was “other,” only “0” was assigned without specifying the cancer type.

All subitems in the nine domains of Section II of the SF-PCTA1.0 were categorized with “reason for referral” and “problem identified by first author” being 1 and “not applicable” being 0. Section II of the SF-PCTA1.0 included nine domains: (1) physical/pharmacological issues, (2) psychiatric/emotional/spiritual issues, (3) diagnosis/anticancer treatment issues, (4) social issues, (5) family issues, (6) place of care, (7) ethical issues, (8) bereaved family issues, and (9) discussion of referral options (Supplementary Data 2). We focused on the first three domains, which were directly related to the patient’s symptoms in Section II of the SF-PCTA1.0. We divided the symptoms into two groups.

We excluded Section III of the SF-PCTA1.0 as input data for the machine learning model because these data were used later than the time of symptom assessment. Section III of the SF-PCTA1.0 included 13 domains: (1) comprehensive assessment, (2) care for patient’s physical symptoms, (3) care for psychiatric symptoms/emotional support for patients, (4) support for patient’s decision making, (5) support for decision making about place of care, (6) support for patient at home, (7) family support, (8) support for ethical issues, (9) referral to specialist, (10) medical procedure/investigation, (11) staff support, (12) coordination within the palliative care team, and (13) pharmacological treatment. The activity items were collected throughout the observation period for each case (Supplementary Data 2).

### Statistical software and analysis flow

We used both Microsoft Excel for Microsoft 365 (Redmond, WA, USA) to prepare the data and RapidMiner (v.9.8.001; RapidMiner, Dortmund, North Rhine-Westphalia, Germany) to create a decision tree. RapidMiner is a flexible Java environment for knowledge discovery in databases, machine learning, and text data mining.

**Figure 2:**
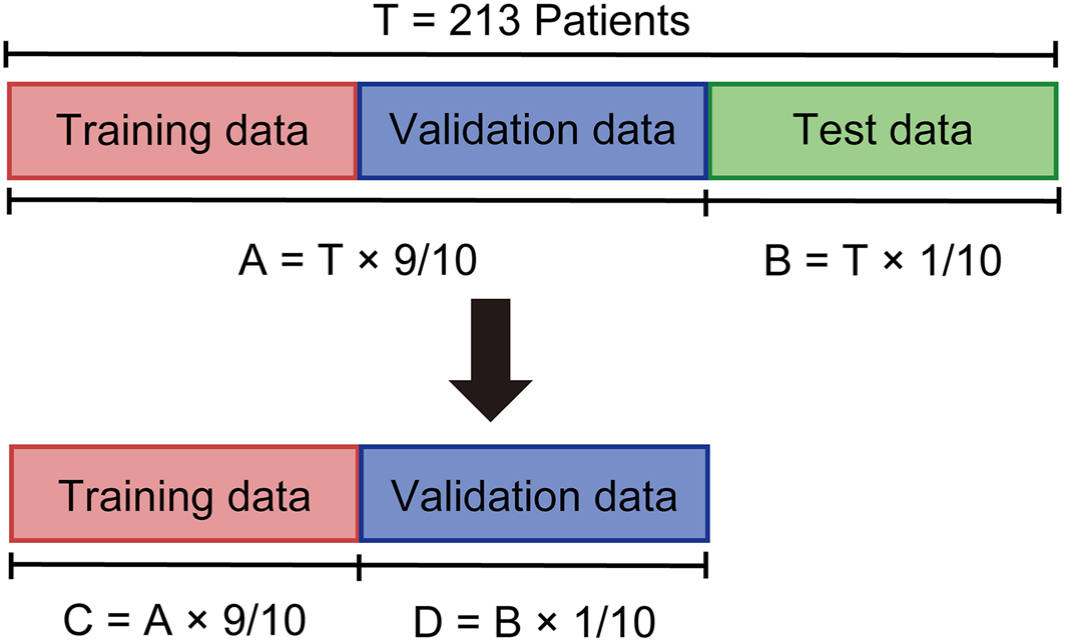
Cross-validation method for learning model creation. (i) 213 patients (= T) were divided into 10 groups that did not overlap with each other, and 10 groups were created by combining 9/10 (= A) and 1/10 (= B). (ii) B in (i) is the test data, and the ten groups did not overlap. Ten sets of A and B were created from the data of the 213 patients. (iii) The A of each group created in (i) was further divided into 10 parts, one for training (= C) and one for validation (= D). (iv) A cross-validation method for randomly generating 10 sets of C and D and a learning model was created on RapidMiner.

**Figure 3:**
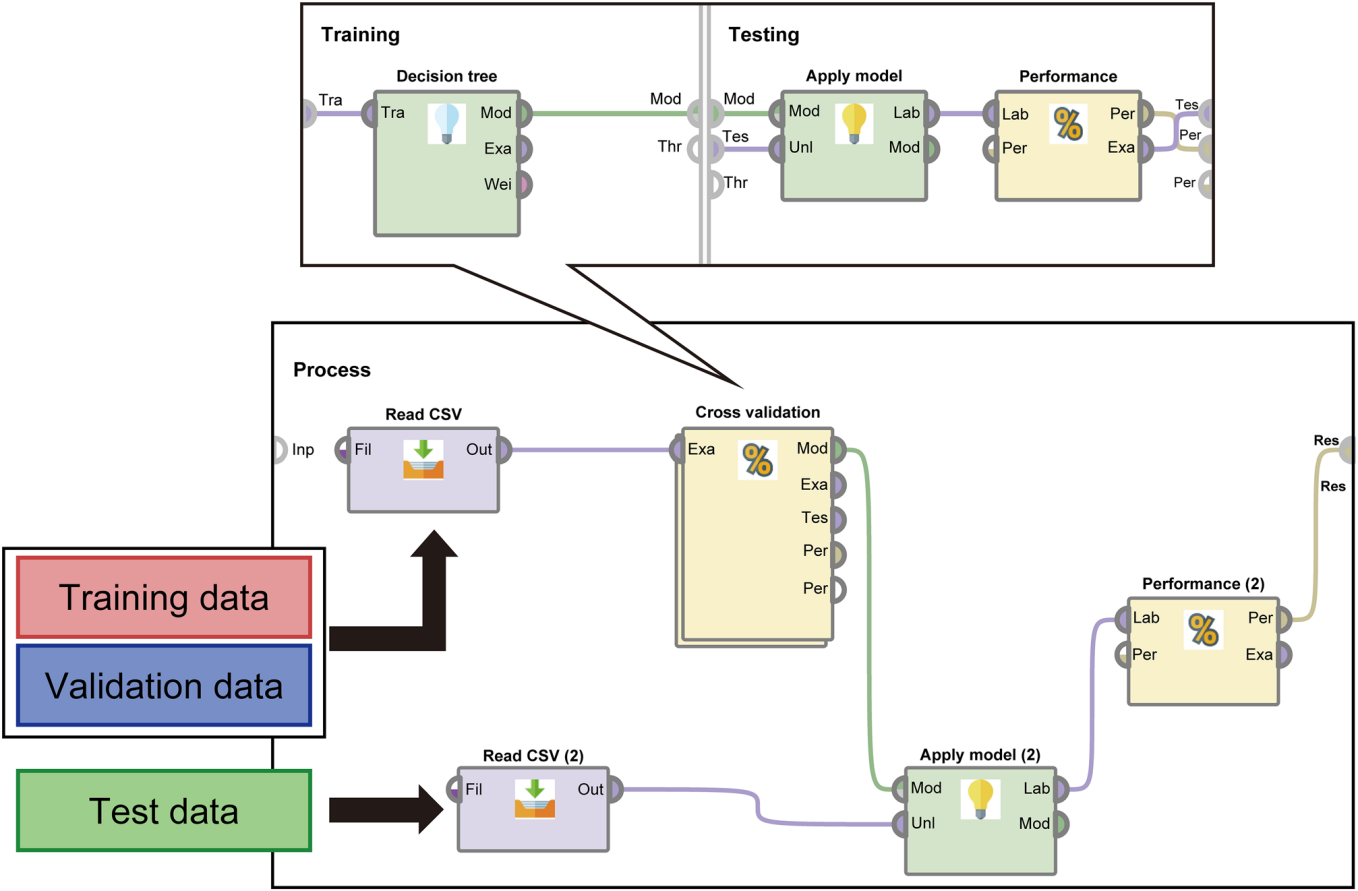
Prediction process for test data. *Tra* training, *Mod* model, *Exa* example, *Wei* weight, *Thr* threshold, *Tes* testing, *Unl* unlabel, *Lab* label, *Per* performance, *Inp* input, *Fil* file, *Out* output, *Res* result.

The analysis procedure using RapidMiner is shown in Figs. 2 and 3. We performed a prediction using the test data in the learning model with a cross-validation method, as shown in Fig. 2. In steps (i)–(ii) shown in Fig. 2, we divided the data of 213 patients into nonoverlapping groups A and B^13^ and created 10 sets of data combinations. Following the general k-split cross-validation method, we set k = 10 because the total number of patients was in units of 100. The dataset was also divided according to the order of the dataset. We used the group B datasets as the test data in each iteration. Next, we developed a learning model from each group A dataset in steps (iii)–(iv) shown in Fig. 2. After the prediction on the 10 sets of test data, as shown in Fig. 3, we used the average of the prediction results of 10 iterations as the final result. Moreover, RapidMiner combines tools called operators to program machine learning, and in this study, we used the decision tree operator (Fig. 3). The gain ratio was used in the decision tree operator, and the random generation of training and validation datasets was specified in the cross-validation operator; however, the other operators and the basic settings of RapidMiner were left at their default values. In this study, we predicted the eight nonvisible symptoms individually rather than simultaneously. We also performed feature extraction to identify the top three features that appeared frequently in the 10 tests from the root node to the leaf node up to and including branch 3 of the decision tree. The frequency of occurrence was set at ≥ 20%, and if no corresponding feature was observed, the features were examined on branch 3 or higher.

Although in collecting patient data, a statistical analysis method was not specified to analyze data from the SF-PCTA1.0 database, our study aimed to create a baseline database to validate various methods for obtaining useful results in clinical practice. Therefore, we used these patient data to create a model using machine learning and classified them as test data to test the machine learning-based model.

Each statistical index was calculated as follows. Sensitivity was defined as true positives (TP)/(TP + false negatives (FN)). Specificity was defined as true negatives (TN)/(TN + false positives [FP]). Accuracy was determined using the following calculation: (TP + TN)/(TP + FP + TN + FN). Finally, we calculated the positive predictive value as TP/(TP + FP) and the negative predictive value (NPV) as TN/(FN + TN).

## Results

Patient characteristics are shown in Table 2. Only adults aged 33–98 years (median age of 69 years) were included, and 53.5% of the patients were male. Various cancer types were included. The total number of cancer centers and university hospitals was 75.6%, which included many patients from hospitals with cancer treatment as their main institutional function. Most patients were inpatients. Although 67.1% of the patients had an ECOG-PS of 3 or higher, 32.8% had an ECOG-PS of 2 or lower, 41.8% received anticancer treatment, and 40.3% had a combined outcome of transfer to a palliative care unit and death. It was estimated that 32.8%–41.8% of the patients were in a good general condition. It is possible that patients may be less able to self-report symptoms if they are in a poor general condition, if they have head and neck cancer with dysphonia, or if they have chemo-brain^14^ complications from chemotherapy. Our goal of creating a machine learning-based model that predicts symptoms difficult to assess with common observation based on patient characteristics and symptoms easy to assess was intended to be able to use cues from symptoms that are valid for observation by others to alert patients of the symptoms that they themselves are unaware of. Because we assumed that the patients’ conditions did not depend on their ability to self-report, we fed all case data into the creation of the machine learning model.

**Table 2:** Background of patients with cancer (n = 213).

Clinical factors	N (%)	
Age (range: 33–98 years, average ± SD: 68.2 ± 13.1 years)		
< 69 years old	102 (47.9)	
≥ 69 years old	111 (52.1)	
Sex		
Male	114 (53.5)	
Female	99 (46.5)	
ECOG^a^ performance status		
1	15 (7.0)	
2	55 (25.8)	
3	81 (38.0)	
4	62 (29.1)	
Types of hospital		
Regional core hospital	52 (24.4)	
Cancer hospital	115 (54.0)	
University hospital	46 (21.6)	
Referring person		
Doctor	75 (35.2)	
Nurse	138 (64.8)	
Place of medical treatment		
Outpatient	15 (7.0)	
Hospitalization	198 (93.0)	
Cancer sites		
Lung	35 (16.4)	
Pancreas	22 (10.3)	
Colon/rectum	20 (9.4)	
Lymph node/hematology	20 (9.4)	
Stomach	18 (8.5)	
Breast	14 (6.6)	
Uterus/ovary	14 (6.6)	
Head and neck	14 (6.6)	
Kidney/bladder	13 (6.1)	
Biliary tract	8 (3.8)	
Other^b^	8 (3.8)	
Liver	7 (3.3)	
Unknown	7 (3.3)	
Prostate	6 (2.8)	
Under investigation	6 (2.8)	
Esophagus	1 (0.5)	
Status of anticancer treatment		
No further anticancer treatment	113 (53.1)	
Under anticancer treatment	89 (41.8)	
Before anticancer treatment	11 (5.2)	
Patient outcome when observation ends		
Observation period ended	67 (31.5)	
Died	71 (33.3)	
Discharge or transfer to	Home	50 (23.5)
	Other	5 (2.3)
	Inpatient hospice/palliative care unit (PCU)	15 (7.0)
Problem resolved	5 (2.3)	
Observation period (range: 1–28 days; average ± SD: 17.5 ± 9.6 days)		
< 18 days	103 (48.4)	
≥ 18 days	110 (51.6)	

The predictive performance of the learning model with decision trees for nonvisible symptoms is shown in Table 3. The symptoms in Table 3 are arranged in the order of predictive accuracy. Drowsiness, which had the highest accuracy, also had the highest specificity among all symptoms. For fatigue, accuracy was third, while the area under the receiver operating characteristic curve was highest among all symptoms. For pain, the accuracy was the fifth highest, while the prediction sensitivity was the highest among nonvisible symptoms.

**Table 3:** Predictive performance of learning models created by decision trees on nonvisible symptoms.

Nonvisible symptoms	Frequency of patients labeled as symptom positive (%)	Sensitivity (%)	Specificity (%)	Accuracy (%)	Area under receiver operating characteristic curve (AUROC)	Positive predictive value (PPV) (%)	Negative predictive value (NPV) (%)
Drowsiness	9.5 ± 7.2	3.3 ± 10.5	96.7 ± 3.9	88.0 ± 8.2	0.450 ± 0.207	10.0 ± 31.6	90.6 ± 7.2
Spiritual issues	21.5 ± 9.4	21.7 ± 35.2	90.8 ± 8.4	74.0 ± 10.7	0.558 ± 0.235	25.0 ± 35.4	79.7 ± 10.9
Fatigue	25.5 ± 9.3	34.5 ± 30.8	88.0 ± 12.0	73.5 ± 6.3	0.706 ± 0.146	48.0 ± 38.9	80.4 ± 9.7
Delirium	19.0 ± 3.2	29.8 ± 20.9	85.7 ± 11.2	71.0 ± 10.2	0.654 ± 0.147	40.2 ± 27.4	78.6 ± 10.7
Pain	70.5 ± 16.4	84.9 ± 7.1	24.1 ± 19.6	68.5 ± 14.2	0.582 ± 0.151	72.9 ± 16.4	37.7 ± 27.4
Dyspnea	27 ± 13.0	27.9 ± 28.1	70.6 ± 13.8	59.5 ± 11.4	0.482 ± 0.175	19.6 ± 20.3	72.3 ± 15.7
Anxiety	52.5 ± 15.1	67.3 ± 12.5	41.8 ± 21.6	56.0 ± 8.1	0.533 ± 0.162	56.8 ± 15.8	51.0 ± 24.7
Inadequate informed consent	38.0 ± 18.9	28.0 ± 16.2	71.2 ± 10.0	55.5 ± 14.6	0.460 ± 0.134	36.6 ± 17.8	62.3 ± 20.5

*TP* true positive, *TPR* true positive rate, *TN* true negative, *FN* false negative, *FP* false positive, *FPR* false positive rate, *ROC* receiver operating characteristic curve.

Sensitivity = TP/(TP + FN).

Specificity = TN/(TN + FP).

Accuracy = (TP + TN)/(TP + FP + TN + FN).

Area under ROC (AUROC) = ${\int }_{x=0}^{1}TPR(FPR{}^{-1}(x))dx$

Positive predictive value (PPV) = TP/(TP + FP).

Negative predictive value (NPV) = TN/(FN + TN).

The aggregated results are shown in Table 4, and constipation and sleep disturbance, both considered visible symptoms, were among the top three features for drowsiness that achieved the highest prediction accuracy. Additionally, two of the top three features for drowsiness were visible symptoms, whereas only one visible symptom, edema, was included in the top three features for fatigue. As with fatigue, only one visible symptom, edema, was included in the top three features for delirium, dyspnea, and anxiety. No visible symptoms were observed in the top three features for spiritual issues, pain, and inadequate informed consent.

**Table 4:** The top three features for predicting nonvisible symptoms extracted by decision trees.

Nonvisible symptoms	Rank	Branch number^a^	Frequency (%)^b^	Features used to predict nonvisible symptoms	
				Clinical factors	Attributes
Drowsiness	1	1	80	Constipation	Visible symptoms
	2	2	70	Age	Background of patients with cancer
	3	2	60	Sleep disturbance	Visible symptoms
Spiritual issues	1	1	80	Age	Background of patients with cancer
	2	4	20	Biliary tract cancer	Background of patients with cancer
	2	4	20	Prostate cancer	Background of patients with cancer
Fatigue	1	1	60	Edema	Visible symptoms
	2	1	20	Cancer of unknown origin	Background of patients with cancer
	3	2	70	Age	Background of patients with cancer
Delirium	1	1	80	Age	Background of patients with cancer
	2	2	80	ECOG performance status^c^	Background of patients with cancer
	3	3	20	Edema	Visible symptoms
Pain	1	1	80	Age	Background of patients with cancer
	2	4	40	Male	Background of patients with cancer
	2	4	40	Biliary tract cancer	Background of patients with cancer
Dyspnea	1	1	70	Age	Background of patients with cancer
	2	2	40	Breast cancer	Background of patients with cancer
	3	3	30	Edema	Visible symptoms
Anxiety	1	1	100	Age	Background of patients with cancer
	2	5	20	Edema	Visible symptoms
	3	8	40	Lymphatic/hematologic cancer	Background of patients with cancer
Inadequate informed consent	1	1	50	Outpatient	Background of patients with cancer
	2	1	20	Under investigation of cancer	Background of patients with cancer
	3	2	60	Breast cancer	Background of patients with cancer

^a^The branch number represents the number of branches from the root node of the tree.

^b^Frequency indicates how often the feature appears in predictions of learning models created by decision trees for test data.

^c^ECOG: Eastern Cooperative Oncology Group.

## Discussion

To the best of our knowledge, this study is the first to predict nonvisible symptoms using decision tree analysis in cancer palliative care. We developed a simple method for predicting nonvisible symptoms from the patient’s background and visible symptoms easy to assess objectively using decision tree analysis, a machine learning algorithm. Recently, research on the clinical applications of machine learning has grown at a remarkable rate. However, most studies were retrospective and theoretical, and only some studies were of sufficient quality to justify costly clinical trials and ongoing quality control as medical devices^15^. Overcoming translational barriers, such as real-time access to clinical data, data security, release of black-box results, and performance evaluation, are considered necessary for the clinical application of machine learning-based predictions^16^. However, we predicted symptoms difficult to assess objectively from symptoms easy to assess, rather than making a diagnosis and predicting the prognosis from images and laboratory data. Our model can advance clinical applications with a simpler system than traditional machine learning studies that use images and molecular biology markers. The global trend of the coronavirus disease 2019 pandemic, which has emphasized the need for telemedicine in times of disaster, is expected to prompt technological advances to support telemedicine^17^. Our model is simple and has potential clinical applications using smartphones and tablets. Because we only need to add a new machine learning model to existing telecommunications technology, the feasibility of social implementation is high, both in terms of technology and cost. In this study, clinical data were retrospectively collected. In addition to the ethical aspects of the clinical data collection, such as the potential harm to patients, the safety of the data was ensured by the fact that the data used by machine learning as the correct answer have been confirmed by experts in palliative care. The SF-PCTA1.0 describes the process of support as part of the medicine team that includes consultation with members of the palliative care team assigned to each facility; therefore, in effect, the accumulated results of multidisciplinary medical care were used as data for machine learning.

Furthermore, the use of the SF-PCTA1.0 allowed us to avoid natural language processing problems, even though the study was conducted with linguistic information on symptoms. Research on the automatic extraction of useful patient information from medical records using natural language processing is still in its infancy and has not yet been applied in actual clinical practice^18^. Few studies have aimed to assess symptoms not found in medical records to help with medical treatment, as in this study. Both in Japan and abroad, symptom assessment tools for patients with cancer are mainly in the form of questionnaires completed by the patients themselves or their healthcare professionals^19^. The application of machine learning in this study has high potential for widespread use in clinical practice because it uses items as input that can be assessed by non-specialists in palliative care.

Moreover, the features extracted by the decision tree analysis can provide clues to the pathophysiology of cancer. Traditionally, in situations where palliative care is more important than anticancer treatment, conducting clinical trials has been difficult because of ethical considerations and the difficulty of adjusting for patient backgrounds^20^. Thus, exploratory basic research on the pathogenesis of various symptoms to serve as a basis for drug development has not been adequately conducted. The nonvisible symptoms in this study can be summarized as the clinical phenotype of abnormalities in involuntary functions of the human body, such as digestion, fluid volume regulation, and sleep. In contrast, the visible symptoms can be summarized as the clinical phenotype of abnormalities in voluntary functions of the human body, such as risk avoidance, exercise tolerance, and state of consciousness. The nervous system can be broadly divided into the peripheral and central nervous systems, with the peripheral nervous system being classified into the somatic and autonomic nervous systems^21^. The somatic nervous system is responsible for collecting sensory input and directing effector organs for voluntary functions. Somatic movements are mediated by the cerebral cortex and higher brain centers in the cerebellum. Meanwhile, the autonomic nervous system controls the effector organs responsible for involuntary homeostatic functions^21^. The nonvisible symptoms in this study may be related to abnormalities in involuntary function—the autonomic nervous system—whereas the visible symptoms may be related to abnormalities in voluntary function, i.e., the somatic nervous system. Therefore, the peripheral nervous system itself and the linkage between the peripheral and central nervous systems are expected to be potential targets for new treatment methods. Various distressing symptoms accumulate during the clinical course of patients with cancer, and methods for predicting the prognosis based on various symptoms are being investigated^22–25^. The fact that visible symptoms were extracted as features in predicting nonvisible symptoms in this study suggests the possibility of predicting central nervous system disorders from autonomic nervous system disorders. Further research is required to determine whether autonomic nervous system disorders are casually related to central nervous system disorders and what mechanisms of these disorders underlie various distressing symptoms in patients with cancer.

The strength of this study is that applications based on our results may be able to assess symptoms to the same extent as healthcare professionals. To determine how much accuracy should be ensured in symptom prediction in the decision tree analysis, we searched for studies on symptom assessment by healthcare professionals in cancer palliative care; however, we found no suitable precedents. Although several studies have examined the frequency of symptoms in patients with cancer^22,26^, no studies have examined indicators that can be used as a reference for how much prediction accuracy by machine learning can withstand clinical application, such as the rate of correct responses to symptom assessment by healthcare professionals. Therefore, we examined the accuracy of symptom prediction by the referring individuals (e.g., physicians and nurses) from the database used in this study (Supplementary Data 3). Because FP results in symptom prediction cannot be accurately confirmed by the referring person, the sensitivity and NPV, which are measures of prediction accuracy and do not include FP, are presented in Supplementary Data 4. For the physical symptoms, drowsiness, fatigue, pain, and dyspnea, and the psychiatric symptoms, delirium and inadequate informed consent, both sensitivity and NPV were better for prediction by the healthcare professionals than for prediction by the decision tree analysis, as can be seen in Table 3 and Supplementary Data 4. In another study predicting patient internalization by objective measures, the primary goal of machine learning was to achieve the same level of accuracy as that in the assessment by healthcare professionals^27^. Additionally, the sensitivity of the decision tree analysis was better than that of the referring person’s ratings for anxiety and spiritual issues. This means that our application may perform better than healthcare professionals in terms of anxiety and spiritual issues. We expected it to be useful in screening symptoms, particularly because of its high sensitivity^28^. Although anxiety has a high prevalence among patients with cancer, this may be overlooked because it rarely occurs in isolation but is combined with physical symptoms, such as dyspnea and fatigue, and sleep disturbance^29^. Spiritual issues have not been adequately evaluated even with conventional questionnaire methods^30,31^ and are also easily overlooked. Therefore, our application may surpass the skills of general healthcare professionals in terms of predicting anxiety and spiritual issues. In the future, empirical research should be conducted to evaluate the performance of the results of this study when applied in clinical practice.

This study also had several limitations. First, this study only included adult patients with cancer. Reports have shown that adults and children show differences in reporting symptoms; therefore, our results may not be valid in children. Second, the number of outpatients included in this study was small; therefore, additional studies should focus on the validity of our model for these patients. Third, our model may not accurately predict future events; thus, further work should investigate this question.

We created a learning model to predict nonvisible symptoms from patient background and visible symptoms, which can be useful as a supportive tool in cancer palliative care. Although the proposed application is unlikely to be an absolute replacement for palliative care specialists, it is expected to help improve the quality of palliative care provided by healthcare professionals. Our results will help better assess and manage symptoms in patients with cancer.

## Supplementary Information

Supplementary Information.

## Data Availability

The individual-level data reported in this study are not publicly available. Individuals wishing to access the disaggregated data, including the specific data reported in this study, should submit a request for access to KS (mobile_pcu@kuhp.kyoto-u.ac.jp). Deidentified data (including, as applicable, participant and relevant data dictionaries) will be shared upon approval of analysis proposals with the signed data access agreements in place.
