# Supplementary material for: Novel method for predicting nonvisible symptoms using machine learning in cancer palliative care
Source: Sci Rep. 2023 Jul 26;13:12088. doi: 10.1038/s41598-023-39119-0 (PMC10371999; doi:10.1038/s41598-023-39119-0)
Supplement: Supplementary file 1 — Supplementary Information. [file 41598_2023_39119_MOESM1_ESM.docx]

**Novel method for predicting nonvisible symptoms using machine learning in cancer palliative care**

Kazuki Shimada^1^* and Satoru Tsuneto^2^

^1^Department of Palliative Medicine, Kyoto University Hospital, 53 Kawaharacho, Shogoin, Sakyo-ku, Kyoto 606-8507, Japan

^2^Graduate School of Medicine, Kyoto University, Department of Palliative Medicine, Kyoto University Hospital, 53 Kawaharacho, Shogoin, Sakyo-ku, Kyoto 606-8507, Japan

***Corresponding author**

Tel: +81-75-751-4629

FAX: +81-75-751-4423

E-mail: mobile_pcu@kuhp.kyoto-u.ac.jp

**Supplementary Data 1. First author’s activities in palliative care consultation**

The first author was transferred from a university hospital to work as a medical adviser in a depopulated area of Fukui Prefecture and worked as a palliative care physician for 1 day each week at a university hospital, a regional core hospital, and a cancer hospital. At that time, the first author was the only palliative medicine specialist in Fukui Prefecture certified by the Japanese Society of Palliative Medicine. The goal was that the first author would compensate for the lack of palliative care in the overall community by serving as a sub-member of the palliative care team of each facility. At each facility, the first author primarily supported the in-house palliative care team and participated in rounds and conferences. The first author provided support to the palliative care team and the doctors and nurses who consulted with the team. Upon request from each facility, the first author also helped in patient consultations and family care in wards and outpatient palliative care clinics and in study sessions on palliative care for medical personnel. Hence, the first author did not target palliative care wards and home care during the activity period, but focused on general wards.

While performing these support activities, the first author collected activity records to improve palliative care consultations in the future. At that time, few reports existed on the activities of a mobile palliative care team that could serve as guidelines for such activities in Japan. The first author examined the methodology of palliative care consultation by evaluating the actual practice of palliative care consultations and trends across different conditions of specialized palliative care at multiple facilities.

**Supplementary Data 2. Response method for standard format for palliative care team activities 1.0 (SF-PCTA1.0) items.**

The content of the SF-PCTA1.0 can be broadly classified into the (i) Cover Sheet, (ii) Reasons for Referral and Initial Assessment, and (iii) Activities. The Cover Sheet includes items related to age, sex, distinction between cancer and noncancer, sites of cancer in the body, anticancer treatment status, Eastern Cooperative Oncology Group Performance Status (ECOG-PS), referring persons, and patient outcomes when the observation ended. While accumulating patient data, the observation period and type of hospital were added to the Cover Sheet. The Reason for Referral and Initial Assessment includes nine areas: (1) physical/pharmacological issues, (2) psychiatric/emotional/spiritual issues, (3) diagnosis/anticancer treatment issues, (4) social issues, (5) family issues, (6) place of care, (7) ethical issues, (8) bereaved family issues, and (9) discussion of referral options. The entries in the Cover Sheet and Reason for Referral and Initial Assessment were completed when support was started. The activities include (1) comprehensive assessment, (2) care for patient’s physical symptoms, (3) care for patient’s psychiatric symptoms/emotional support, (4) support for patient’s decision making, (5) support for decision making about the place of care, (6) support for the patient at home, (7) family support, (8) support for ethical issues, (9) referral to a specialist, (10) medical procedure/investigation, (11) staff support, (12) coordination within the palliative care team, and (13) pharmacological treatment. The activity items were completed throughout the observation period for each case. During the initial assessment, each subitem was clustered into the following: “Reason for Referral,” “Problem Identified,” and “Not Applicable.” The “Reason for Referral” is the reason why the healthcare professionals at the target institutions requested case consultations from the first author. The “Problem Identified” is the problem raised by the first author to the medical staff at the target facility during the initial consultation. Items that were not requested or identified were marked “Not Applicable.” The contents of activity were clustered by subitems as follows: “Recommended,” “Performed,” or “Not Applicable.” “Recommended” indicates that a proposal was made to the client. “Performed” indicates that the first author personally implemented the activity. Items not recommended or implemented were classified as “Not Applicable.” If no lowest-level item existed in the initial evaluation or activity content, the items were accumulated using one higher-level item. The actual form used by the authors is shown in the Appendix.

# Appendix

*Standard Format for Reporting Hospital Palliative Care Team Activities 1.0 (SF-PCTA1.0).*

- 1. Cover Sheet

| Date of referral (dd/mm/yy) | / / |  |  |
| --- | --- | --- | --- |
| Age | ( ) years old |  |  |
| Sex | ,Male | ,Female |  |
| Diagnosis | ,Cancer | ,Noncancer |  |
| Cancer site | ,Lung | ,Esophagus | ,Stomach |
|  | ,Colon/rectum | ,Liver | ,Pancreas |
|  | ,Biliary tract | ,Breast | ,Uterus/ovary |
|  | ,Kidney/bladder | ,Prostate | ,Head and neck |
|  | ,Central nervous system | ,Lymph node/hematology | ,Unknown |
|  | ,Under investigation | ,Other |  |

Status of anticancer treatment ,No further anticancer treatment

,Under anticancer treatment

,Before anticancer treatment

ECOG Performance Status ,0, 1, 2, 3, 4

Referring person ,Doctor, Nurse, Other Patient outcome when observation ends Discharge or transfer to

,Home

,Inpatient hospice/palliative care unit (PCU)

,Other

,Observation period ended

,Died

,Problem resolved

ECOG ¼ Eastern Cooperative Oncology Group.

1. Reason for Referral and Initial Assessment

Category Item

Physical/pharmacological issues Pain

Appetite loss/difficulty in oral intake Dyspnea/cough/sputum

Fatigue Nausea/vomiting

Abdominal swelling/ascites Constipation Edema/lymphedema Drowsiness

Oral problem

Present symptom palliation review

Choice of drugs/change in the drug dosage or the route of administration

Other

Psychiatric/emotional/spiritual issues Anxiety/depression/grief/emotional distress

Insomnia Delirium Spiritual issues Other

Diagnosis/anticancer treatment issues Illness understanding/choice of treatment

Anxiety about side effects of anticancer treatment Communication difficulties with clinical staff

Social issues Economic/work problem Absence of a caregiver

Family issues Anxiety/depression/grief/emotional burden Illness understanding/choice of treatment Shortage of practical knowledge/skills

Place of care Ethical issues

Bereaved family issues

Discussion of referral options Establishing rapport between patient and PCT

in preparation for future deterioration

Other

Reason for Referral

Problem Identified by Palliative Care Team (PCT)

1. Activity

Component Category Detailed Item Recommended Performed

1. Comprehensive assessment
2. Care for patient’s physical symptoms

Identify what the patient worries about the most Assess patient’s physical symptoms

Assess patient’s psychological status Assess patient’s understanding of illness

Patient education about opioids Educate how to use opioids effectively

Address anxiety about using opioids

Help to prevent physical distress Any of the following: increasing or decreasing number of body

position changes, using or changing devices for comfort, devising a way for transferring, changing or designing diet, encouraging or designing oral care

Care for pain Any of the following: comprehensive assessment, setting a treatment goal, identifying exacerbating or ameliorating factors, introducing a method for symptom palliation matching patient’s and family’s needs, educating how to address worsening symptoms/educating how to prevent symptom exacerbation

Care for dyspnea

Care for nausea/vomiting/appetite loss

Care for abdominal swelling/ascites Care for dry mouth

Care for fatigue

Care for edema/lymph edema Care for drowsiness

1. Care for psychiatric symptoms/emotional support for patients

Care for delirium Care for insomnia

General psychiatric support Establish relationship with patient

Help patient accept reality of their condition Help patient accept their feelings

Enhance social support

Provide relaxing environment

Individual care Enhance relationships with family

Enhance patient’s sense of physical control Enhance patient’s sense of control of the future Help patient maintain their identity

Help maintain patient’s hope

Relieve the sense of burden to others Help to complete unfinished business Relieve anxiety about death

1. Support for patient’s decision making
2. Support for decision making about place of care

Confirmation of preference and coordination

Assess for insight and preference of disclosure

Provide additional information to support decision making

Coordinate additional information for patients Bridge thoughts between all involved persons

Confirm patient’s preference and coordinate Confirm family’s preference and coordinate

1. Support for patient at home
2. Family support
3. Support for ethical issues
4. Referral to specialist

Support when discharged Any of the following: assessing environment at home, changing or coordinating medical procedures to those that are easily used at home, referring to community health services, addressing when patient has distress at home, confirming contact person or section after discharge

Support when transferred to PCU Judge whether the timing of transfer to PCU is

appropriate or not/coordinate Refer to PCU

Any of the following: monitoring or addressing patient at home, reporting the results of monitoring to physicians in outpatient services, providing advice for community healthcare professionals

Comprehensive assessment Identify what the family worries about the most

Emotional support Assess family’s psychological status

Accept the feelings of the family Give advice on how to treat the patient

Explain the dying process

Support for decision making Any of the following: assessing for the preference of disclosure, providing additional explanation to support decision making, coordinating for patients to have additional explanation, bridging thoughts between all involved persons

Care for family’s burden Assess family’s burden

Coordinate care services for the patient

Specialist in the psychological field Radiotherapist, medical oncologist, surgeon, orthopedist, pain clinician/anesthesiologist, discharge section/medical social worker, rehabilitation, others (nutrition support team, wound, ostomy, and continence nurse and dental hygienist)

(*Continued*)

III. Continued

Component Category Detailed Item Recommended Performed

1. Medical procedure/investigation

Drainage Pleural effusion, ascites

Artificial hydration Reducing the amount, withdrawing, changing the types of hydration

Nerve block Nerve block, TENS, acupuncture

Investigations Blood tests, X-ray, CT, MRI, bone scintigraphy, PET

Other Initiating blood transfusion or oxygen, using prosthetics, initiating or withdrawing inhalation, initiating or withdrawing suction

1. Staff support

Comprehensive assessment Identify what the referring staff feels is difficult Clarify underlying problems

Sharing information among staff in an institution

Sharing information among staff in a community

Confirm whether information is shared Coordinate individually to encourage sharing

information

Hold a conference for sharing information

Any of the following: confirming whether information is shared, coordinating individually to encourage sharing information, or holding a conference for sharing information

Education of staff Explain to the staff what the patient’s distress is, the extent of the patient’s distress, and the cause of the patient’s distress

Explain to the staff how we can palliate the patient’s distress

Emotional support for staff Provide positive feedback about the treatment/care that the staff provided

Accept staff feelings

1. Coordination within PCT
2. Pharmacological treatment

Assessment for institution, sections, staff

Assessment of the characteristics, competency, and function of the institution, sections, and staff

Organize in preparation for when PCT members are unavailable

Assess and coordinate work balance of the team members of the PCT

Analgesics Non-analgesics New administration Adjustment

Opioids (regular) New administration Adjustment

Opioids (rescue) New administration Adjustment

Adjuvant analgesics New administration Adjustment

Antiemetics Prokinetic agents New administration Adjustment

Antidopaminergics,

antihistaminergics

New administration

Adjustment

Psychoactive drug Anxiolytics New administration Adjustment

Antipsychotics New administration Adjustment

Antidepressants New administration Adjustment

Corticosteroids New administration

Adjustment

Gastrointestinal agents Laxatives New administration Adjustment

Gastric secretion inhibitors New administration Anticholinergics Scopolamines New administration

Others New administration

Adjustment

TENS ¼ transcutaneous electrical nerve stimulation; MRI ¼ magnetic resonance imaging; PET ¼ positron emission tomography.

**Supplementary Data 3. Association between institutional information about specialized cancer care and the frequency of “Reason for Referral” and “Problem Identified” by the first author during symptom assessment.**

| **Symptom assessment** | | **Institutional information on specialized cancer care*^1^** | | **p*^2^** |
| --- | --- | --- | --- | --- |
|  |  | No | Yes |  |
|  |  | N (%) | |  |
| Decrease in food intake | Reason for Referral | 11 (91.7) | 27 (75.0) | 0.414 |
|  | Problem Identified | 1 (8.33) | 9 (25.0) |  |
| Nausea | Reason for Referral | 2 (66.7) | 21 (72.4) | 1.00 |
|  | Problem Identified | 1 (33.3) | 8 (27.6) |  |
| Abdominal distension | Reason for Referral | 6 (75.0) | 14 (66.7) | 1.00 |
|  | Problem Identified | 2 (25.0) | 7 (33.3) |  |
| Constipation | Reason for Referral | 0 (0.00) | 2 (8.70) | 1.00 |
|  | Problem Identified | 5 (100) | 21 (91.3) |  |
| Edema | Reason for Referral | 4 (80.0) | 4 (66.7) | 1.00 |
|  | Problem Identified | 1 (20.0) | 2 (33.3) |  |
| Sleep disturbance | Reason for Referral | 5 (26.3) | 10 (17.2) | 0.505 |
|  | Problem Identified | 14 (73.7) | 48 (82.8) |  |
| Drowsiness | Reason for Referral | 3 (100) | 9 (52.9) | 0.242 |
|  | Problem Identified | 0 (0.00) | 8 (47.1) |  |
| Spiritual issues | Reason for Referral | 1 (6.67) | 5 (17.2) | 0.647 |
|  | Problem Identified | 14 (93.3) | 24 (82.8) |  |
| Fatigue | Reason for Referral | 10 (76.9) | 27 (73.0) | 1.00 |
|  | Problem Identified | 3 (23.1) | 10 (27.0) |  |
| Delirium | Reason for Referral | 5 (45.5) | 19 (47.5) | 1.00 |
|  | Problem Identified | 6 (54.5) | 21 (52.5) |  |
| Pain | Reason for Referral | 22 (91.7) | 117 (93.6) | 0.664 |
|  | Problem Identified | 2 (8.33) | 8 (6.4) |  |
| Dyspnea | Reason for Referral | 12 (75.0) | 26 (59.1) | 0.367 |
|  | Problem Identified | 4 (25.0) | 18 (40.9) |  |
| Anxiety | Reason for Referral | 13 (41.9) | 23 (29.1) | 0.259 |
|  | Problem Identified | 18 (58.1) | 56 (70.9) |  |
| Inadequate informed consent | Reason for Referral | 12 (60.0) | 23 (38.3) | 0.120 |
|  | Problem Identified | 8 (40.0) | 37 (61.7) |  |

*1 Institution not specialized in cancer care: Sugita Genpaku Memorial Obama Municipal Hospital. Institutions that specialized in cancer care: University of Fukui Hospital and Fukui Prefectural Hospital.

*2 Fisher’s exact test.

**Supplementary Data 4. Results of symptom prediction by healthcare professionals (n = 213).**

| **Symptoms** | **Frequency of patients labeled as symptom positive (%)** | **Sensitivity (%)** | **Negative predictive value (NPV) (%)** |
| --- | --- | --- | --- |
| Drowsiness | 9.4 | 63.6 | 96.0 |
| Spiritual issues | 20.7 | 15.6 | 81.6 |
| Fatigue | 23.5 | 78.0 | 92.2 |
| Delirium | 23.9 | 52.6 | 85.2 |
| Pain | 70.0 | 93.9 | 82.8 |
| Dyspnea | 28.2 | 66.7 | 87.0 |
| Anxiety | 51.6 | 33.3 | 58.0 |
| Inadequate informed consent | 32.9 | 43.8 | 74.7 |

True positive is defined as a symptom assessed by both a referring person and a palliative care professional; false negative is defined as a symptom assessed by a palliative care professional but not by a referring person; false positive (FP) is defined as a symptom assessed by a referring person but not by a palliative care professional; and true negative is defined as a lack of symptoms assessed by both a referring person and a palliative care professional. We were unable to exactly calculate FP because the SF-PTCA1.0 did not accurately document that a palliative care professional had noted an overestimation of the referring person’s assessment of symptoms. Because of the element of assumption in FP from the data structure of the SF-PCTA1.0, evaluation indices that include FP as a factor, including accuracy, specificity, area under the ROC (AUC), and positive predictive value, were excluded from Supplementary Data 1.
